# A novel Alzheimer’s disease drug candidate targeting inflammation and fatty acid metabolism

**DOI:** 10.1186/s13195-017-0277-3

**Published:** 2017-07-14

**Authors:** Daniel Daugherty, Joshua Goldberg, Wolfgang Fischer, Richard Dargusch, Pamela Maher, David Schubert

**Affiliations:** Cellular Neuroendocrinology Laboratory, The Salk Institute for Biological Studies, 10010 North Torrey Pines Road, La Jolla, CA 92037-1002 USA

**Keywords:** Alzheimer’s disease, Drug candidate, Gene expression, Metabolomics, Bioinformatics

## Abstract

**Background:**

CAD-31 is an Alzheimer’s disease (AD) drug candidate that was selected on the basis of its ability to stimulate the replication of human embryonic stem cell-derived neural precursor cells as well as in APPswe/PS1ΔE9 AD mice. To move CAD-31 toward the clinic, experiments were undertaken to determine its neuroprotective and pharmacological properties, as well as to assay its therapeutic efficacy in a rigorous mouse model of AD.

**Results:**

CAD-31 has potent neuroprotective properties in six distinct nerve cell assays that mimic toxicities observed in the old brain. Pharmacological and preliminary toxicological studies show that CAD-31 is brain-penetrant and likely safe. When fed to old, symptomatic APPswe/PS1ΔE9 AD mice starting at 10 months of age for 3 additional months in a therapeutic model of the disease, there was a reduction in the memory deficit and brain inflammation, as well as an increase in the expression of synaptic proteins. Small-molecule metabolic data from the brain and plasma showed that the major effect of CAD-31 is centered on fatty acid metabolism and inflammation. Pathway analysis of gene expression data showed that CAD-31 had major effects on synapse formation and AD energy metabolic pathways.

**Conclusions:**

All of the multiple physiological effects of CAD-31 were favorable in the context of preventing some of the toxic events in old age-associated neurodegenerative diseases.

**Electronic supplementary material:**

The online version of this article (doi:10.1186/s13195-017-0277-3) contains supplementary material, which is available to authorized users.

## Background

There are no drugs that prevent the death of nerve cells in Alzheimer’s disease (AD). Because age is the greatest risk factor for AD, we developed a drug discovery paradigm that is based upon phenotypic screens against old age-associated brain pathologies without requiring preselected molecular targets [1, 2]. Six cell culture assays were designed to mimic multiple old age-associated toxicities, and drug candidates were selected that show efficacy in all of these assays [3]. On the basis of these toxicity assays, we identified an exceptionally potent, orally active, neurotrophic molecule called *J147*. J147 facilitates memory in normal rodents, and it prevents the loss of synaptic proteins and cognitive decline when administered to 3-month-old APPswe/PS1∆E9 mice for 7 months [1, 2, 4], as well as in rapidly aging senescence-accelerated mouse-prone 8 (SAMP8) mice, a model of sporadic AD [5]. It also reverses memory deficits and some AD pathology when fed to very old transgenic AD mice [2].

We recently synthesized a derivative of J147 called *CAD-31* that has enhanced neurogenic activity over J147 in human neural precursor cells (NPCs). CAD-31 also stimulates the division of NPCs in the subventricular zone of old APPswe/PS1ΔE9 mice when fed starting from an early age, a preventive strategy [4]. However, the neuroprotective properties of CAD-31 have not been well characterized, and CAD-31 needed to be tested for disease modification in a more relevant model of AD.

To more closely mimic the clinical setting, we examined the effect of CAD-31 in transgenic mice at a stage when pathology is significantly advanced and asked if the drug could rescue AD-associated deficits. APPswe/PS1∆E9 mice exhibit a subset of behavioral and pathological features of AD, including age-dependent accumulation of β-amyloid (Aβ) as well as learning and memory deficits at 10 months of age [1, 6, 7]. These mice were previously used to demonstrate the neurogenic and neuroprotective and memory-enhancing effects of CAD-31 in a preventive paradigm in which CAD-31 was administered before pathology was present [4]. In contrast, the APPswe/PS1∆E9 AD mice in this study were allowed to age to 10 months before being fed CAD-31 for 3 months. Here we show that under these conditions, CAD-31 normalized cognitive skills to those of age-matched wild-type (WT) mice, reduced markers of inflammation and synaptic loss, and shifted the metabolic profile of fatty acids toward the production of ketone bodies, a potent source of energy in the brain when glucose levels are low.

## Methods

### Materials

High-glucose DMEM and fetal calf serum were obtained from Invitrogen (Carlsbad, CA, USA). C57BL/6J mice were ordered from The Jackson Laboratory (stock number 000664; The Jackson Laboratory, Bar Harbor, ME, USA). The transgenic mouse APPswe/PS1∆E9, line 85, was a generous gift of Dr. J. L. Jankowsky (Department of Neuroscience, Baylor College of Medicine, Houston, TX, USA). The primary rabbit antibodies were used at a dilution of 1:1000 unless otherwise stated, and their sources were as follows: β-actin, mouse monoclonal HRP conjugate; voltage-dependent anion channel; Arc-1; clusterin; phospho-S51-eukaryotic initiation factor 2α (eIF2α) and total eIF2α; ubiquitin; adenosine monophosphate-activated protein kinase (AMPK); phosphor-S72-AMPK; vascular cell adhesion molecule (VCAM); receptor for advanced glycation endproducts (RAGE); oligomycin sensitivity-conferring protein (OSCP); doublecortin (DCX); drebrin; and phospho-S79-acetyl-coenzyme A carboxylase 1 (ACC-1) (all from Cell Signaling Technology, Danvers, MA, USA). All other materials were purchased from Sigma-Aldrich (St. Louis, MO, USA) unless otherwise stated.

### Phenotypic screening assays

The various phenotypic screening assays were conducted as previously described [3]. Briefly, HT-22, primary cortical neurons, or MC65 was plated and exposed to the different environmental stresses. Cells were treated with varying concentrations of CAD-31, and half-maximal effective concentration (EC_50_) was determined on the basis of cell viability.

### Animal studies

All animal studies were carried out in strict accordance with the recommendations in the Guide for the Care and Use of Laboratory Animals of the National Institutes of Health. The protocol was approved by the Committee on the Ethics of Animal Experiments of the Salk Institute for Biological Studies. The number of mice per group was determined by a power analysis based upon published data from our laboratories and others using this strain of mice.

#### APPswe/PS1ΔE9-transgenic mice

The APPswe/PS1∆E9-transgenic mice (line 85) were characterized previously [7]. Line 85 mice carry two transgenes, the mouse/human chimeric APPswe, linked to Swedish familial AD and human PS1∆E9. At 10 months of age, female transgenic mice were fed a defined diet (Harlan Teklad; Envigo, Indianapolis, IN, USA) with and without CAD-31 (200 ppm, approximately 10 mg/kg/day). Treatment continued for 3 months, followed by behavior testing and tissue harvesting. Mouse body weights and food consumption were measured weekly, and there were no significant differences between the groups (data not shown).

#### Behavior assays

##### Two-day water maze

Spatial memory was determined using the 2-day water maze with 13-month-old APPswe/PS1∆E9 transgenic mice fed CAD-31 for the previous 3 months. The protocol was adapted from a publication by Gulinello and colleagues [8]. The goal platform was positioned 45 cm from the outside wall in the northwest quadrant of the maze. Day 1 involved training the mice (*n* = 10 per group) to find the platform using cues located around the pool within a 180-second time frame. This training involved a series of visible platform trials where mice were tracked using EthoVision software (Noldus Information Technology, Leesburg, VA, USA). There were four visible platform trials (V1–V4) where the last visible platform trial of a mouse was considered its posthabituation baseline. If the mice failed to find the platform after 180 seconds, they were placed on the platform by the experimenter. All mice remained on the platform for 15 seconds before being placed in a heated incubator (30 °C) between trials. On day 2, 24 h following the last visible platform trial, mice were tested in a hidden platform trial (T1). The trial lasted for 180 seconds. The time it took each mouse to find the hidden platform was measured as escape latency. All trials were recorded using the EthoVision software, and statistics were computed using InStat software (GraphPad Software, La Jolla, CA, USA) by an individual blinded to the experiment.

##### Elevated plus maze

The elevated plus maze is used to analyze the anxiety response of mice [9]. This test relies upon the tendency of mice to have a fear of heights and to navigate toward dark, enclosed spaces and remain there [10]. Our maze is made of gray plastic and consists of four arms (two open without walls and two enclosed by 15.25-cm-high walls) that are 30 cm long and 5 cm wide in the shape of a plus sign. A video tracking system (EthoVision; Noldus Information Technology) is used to automatically collect behavioral data. Mice are habituated to the room 24 h before testing. Mice are also habituated to the maze for 2 minutes before testing by placing them in the center of the maze and blocking entry to the arms. Mice were tested in the maze for a 5-minute period. The anxiety of mice was measured by comparing the time spent in the open arms with time spent in the closed arms. Statistics were computed using GraphPad InStat software.

##### Fear-conditioning assay

Fear conditioning to either a cue or a context represents a form of associative learning. The readout that is measured in contextual and cued fear conditioning is a freezing response that occurs following the pairing of an unconditioned stimulus, such as a foot shock, with a conditioned stimulus, such as a particular context or cue (tone) [11–13]. The mouse will freeze if it remembers and associates that environment with the aversive stimulus. The hippocampus and the amygdala are required for fear memory, where the hippocampus is involved in the formation and retrieval of context fear associations and the amygdala is involved in conditioning and recall of associations to contextual and discrete cues [14, 15]. With this assay, we used fear-conditioning chambers from Med Associates Inc. (Fairfax, VT, USA) with freeze monitoring software. On day 1, mice (*n* = 10 per group) were trained by allowing them to explore the chamber for 120 seconds, then presented with a 30-second tone (2 kHz with 85-dB intensity) immediately, followed by a 2-second foot shock (0.7 mA). The tone-shock pairing was repeated following a 30-second interval, and the mice were again allowed to explore for 120 seconds before being removed from the chamber. On day 2, contextual memory, which requires a functioning hippocampus, was tested by placing the mice in the chambers and allowing them to explore for the same length of time as the previous day, but without the tone and the shock. The camera measures the amount of time the mice freeze, and the software allows analysis of this freezing at any time point of interest. On day 2, the time spent freezing is measured over the entire time in the chamber. A mouse that remembers the chamber context and associates it with the foot shock will spend more time freezing, and this response is hippocampus-dependent. The percentage of time spent freezing by each mouse is averaged per group, and then groups can be compared and *p* values calculated to determine statistical significance.

### Tissue preparation and immunoblotting

Hippocampal tissue samples were homogenized in 10 volumes of radioimmunoprecipitation assay (RIPA) lysis buffer (50 mM Tris, pH 7.5, 150 mM NaCl, 0.1% SDS, 0.5% deoxycholate, and 1% Nonidet P-40) containing a cocktail of protease and phosphatase inhibitors [20 mg/ml each of pepstatin A, aprotinin, phosphoramidon, and leupeptin; 0.5 mM 4-(2-aminoethyl)benzenesulfonyl fluoride hydrochloride; 1 mM ethylene glycol-bis(β-aminoethyl ether)-*N,N,N′,N′*-tetraacetic acid; 5 mM fenvalerate; and 5 mM cantharidin]. Samples were sonicated (2 × 10 seconds) and centrifuged first at 10,000 × *g* for 10 minutes and then at 100,000 × *g* for 60 minutes at 4 °C. The 100,000 × *g* pellet was taken up either in 6 M guanidine for Aβ analysis or in SDS sample buffer for Western blot analysis. Protein concentrations in the cell extracts were determined using a bicinchoninic acid protein assay (Pierce Biotechnology, Rockford, IL, USA). Equal amounts of protein were solubilized in 2.5× SDS sample buffer, separated on 12% SDS-polyacrylamide gels, transferred to Immobilon-P (EMD Millipore, Billerica, MA, USA), and immunoblotted with the antibodies indicated in the Materials subsection above. For Western blot experiments, protein levels were normalized to actin levels. An unpaired *t* test was performed to compare two groups at a single time point. When comparing multiple groups, one-way analysis of variance (ANOVA) followed by Tukey’s post hoc test was used. All statistical analysis was conducted using GraphPad InStat software.

### Aβ enzyme-linked immunosorbent assay

Aβ_1–42_ levels in hippocampal lysate were analyzed using Aβ_1–42_ enzyme-linked immunosorbent assay kits from Invitrogen (catalogue number KHB3442). All kit reagents were brought to room temperature before use. Standards were prepared according to the manufacturer’s guidelines, and samples were diluted as follows; RIPA fractions were diluted 1:10 for Aβ_1–42_, and RIPA insoluble fractions were diluted 1:5000 for Aβ_1–42_. A quantity of 50 μl of Aβ peptide standards and samples was added in duplicate to 96-well plates precoated with antibody to the NH_2_-terminal region of Aβ. Plates were incubated at 4 °C overnight, and then 50 μl of human Aβ_42_ detection antibody was added to each well except the chromogen blanks. Plates were incubated at room temperature with gentle shaking for 3 h and then washed four times with the provided wash buffer. At that time, 100 μl of antirabbit immunoglobulin G HRP working solution was added to each well except the chromogen blanks for 30 minutes at room temperature. Wells were then washed as before four times and incubated with 100 μl of stabilized chromogen for 25 minutes at room temperature in the dark. Stop solution was then added at 100 μl to each well, followed by reading the absorbance of each well at 450 nm. Curve-fitting software was used to generate the standard curve where a four-parameter algorithm provided the best standard curve fit. The concentrations of the samples were calculated from the standard curve and multiplied by the dilution factor.

### Pharmacokinetics and free feeding CAD-31 assay protocols

Sprague-Dawley rats had free access to food and water. CAD-31 was given by gavage to rats at 20 mg/kg in corn oil and intravenously in 15% HS 15/PBS. Whole-blood samples were collected from the jugular vein at every time point and brain collected after 20-ml saline perfusion. CAD-31 was extracted with acetonitrile and compared with standards on a TSQ Quantiva™ Triple Quadrupole Mass Spectrometer (Thermo Scientific, Waltham, MA, USA). For the mouse feeding studies, mice had free access to food containing 200 ppm CAD-31. Blood was collected by heart puncture, and the brain concentration was determined as with rats.

### Whole-transcriptome RNA-sequencing analysis

RNA was isolated from the hippocampus using the RNeasy Plus Universal Mini Kit (QIAGEN, Valencia, CA, USA). RNA sequencing (RNA-seq) libraries were prepared using the TruSeq Stranded mRNA Sample Prep Kit (Illumina, San Diego, CA, USA) according to the manufacturer’s instructions. Briefly, polyadenylation RNA was selected using oligo(dT) beads. Messenger RNA was then fragmented and reverse-transcribed. Complementary DNA (cDNA) was end-repaired, adenylated, and ligated with Illumina adapters with indexes. Adapter-ligated cDNA was then amplified. Libraries were pooled and sequenced single-end 50 bp on the HiSeq 2500 platform (Illumina). Sequencing reads were quality-tested using FastQC and aligned to the mm10 mouse genome using STAR Aligner version 2.4k. Mapping was carried out using default parameters (up to ten mismatches per read and up to nine multimapping locations per read). Raw gene expression was quantified across all gene exons by RNA-seq using the top-expressed isoform as a proxy for gene expression, and differential gene expression was carried out using the edge R package version 3.6.8, using replicates to compute within-group dispersion. *p* Values are adjusted to control the false discovery rate (FDR) by the method of Benjamini and Hochberg. Differentially expressed genes were defined as having an FDR <0.05 and a log2 fold change >1. Further statistical analysis was carried out using AltAnalyze software (http://www.altanalyze.org). Pathway analysis was conducted using the DAVID Bioinformatics Resource 6.8 [16].

### Metabolomic analysis

Metabolomic analyses were conducted at Metabolon (Durham, NC, USA) as described previously [5]. For statistical analyses and data display, any missing values were assumed to be below the limits of detection and imputed with the compound minimum (minimum value imputation). An estimate of the FDR (*Q* value) was calculated to take into account the multiple comparisons that normally occur in metabolomics-based studies, with *Q* < 0.05 used as an indication of high confidence in a result. The MetaboAnalyst tool was used to generate heat maps.

## Results

**Fig. 1:**
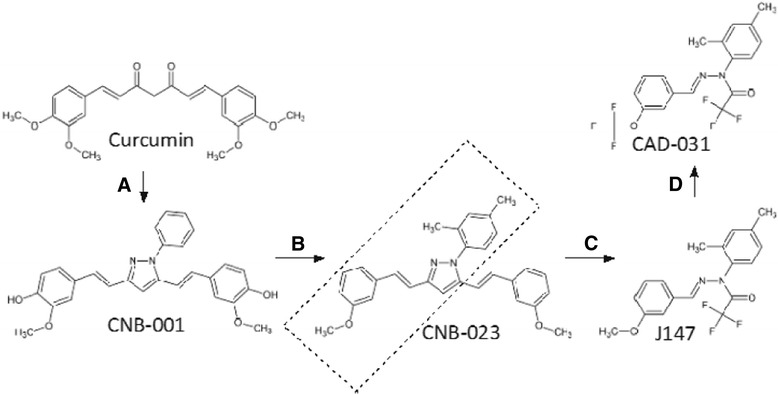
Synthetic origin of CAD-31. CAD-31 was derived from curcumin by following a series of synthetic steps described in the text and outlined here. The selections of CNB-001, CNB-023, and J147 (steps *A*, *B*, and *C* in the figure) were all done on the basis of improved potency in the neurotoxicity assays shown in Table 1. The final step (*D*) needed to generate CAD-31 was done by selecting the most potent compound in a 230-compound J147 library using a human cell-based neurogenesis assay [4]

Figure 1 shows the chemical structure of J147 and CAD-31, as well as how it was sequentially derived from curcumin by an initial structural hybrid with the neurotrophic compound cyclohexyl bisphenol A to yield CNB-001 (step A) [17]. Structural activity relationship-driven chemistry was used to improve the potency of CNB-001 to yield CNB-023 (step B). We then made a large number of structures related to CNB-023, and J147 was identified on the basis of exceptional potency (step C) [1]. CAD-31 was selected for neurogenesis using human embryonic stem cell (ES)-derived NPCs (step D) [1, 4, 17]. The following paragraphs describe the neuroprotective properties of CAD-31, its efficacy in a therapeutic AD model, and its effects on the metabolism of 13-month-old mice.

### CAD-31 is broadly neuroprotective

Because J147 was selected on the basis of its broad neuroprotective activity [1] and CAD-31 was selected on the basis of its neurogenic properties in human cells [4], we asked if CAD-31 maintained all of the neuroprotective activities of J147. Table 1 shows that CAD-31 is biologically active in six assays that represent distinct neurotoxicity pathways related to aging and neurodegenerative disease. CAD-31 is broadly neuroprotective. CAD-31 and J147 were run at the same time in each assay listed below:

**Table 1:** Half-maximal effective concentrations of J147 and CAD-31 in six assays

Assay	Cell type	J147 (nM)	CAD-31 (nM)
Trophic factor withdrawal	Cortical neuron	35	18
BDNF-like activity	HT22	74	95
Oxytosis	Cortical neuron	6	20
In vitro ischemia	HT22	14	47
Aβ toxicity (extracellular)	Hippocampal neuron	15	27
Aβ toxicity (intracellular)	MC65	10	12

*Aβ* β-Amyloid, *BDNF* Brain-derived neurotrophic factor

1. An assay that mimics the dramatic loss of neurotrophic support in the old brain is the trophic factor withdrawal assay [18, 19]. Primary embryonic day 18 cortical cells are plated at low density in serum-free medium. At low density, the cells die within 2 days; they can be rescued by combinations of neurotrophic growth factors, but not by one alone ([19] and unpublished data). Cell death is prevented by both J147 and CAD-31 with EC_50_ of 35 nM and 18 nM, respectively.
2. In the second assay, J147 and CAD-31 are able to rescue HT22 cells expressing the brain-derived neurotrophic factor (BDNF) receptor transmembrane receptor kinase B from serum starvation under conditions where the cells can be protected by BDNF [20]. BDNF is a molecule that is also involved in promoting memory and neurogenesis and is reduced in the brain with age and in AD, as well as in other neurological disorders [21, 22]. BDNF has long been considered a drug target for AD [23]. CAD-31 was slightly less potent in this assay, with an EC_50_ of 95 nM vs 74 nM for J147.
3. J147 and CAD-31 rescue primary cortical neurons from oxytosis, an oxidative stress-induced programmed cell death pathway initiated by glutathione (GSH) depletion [24]. A reduction in GSH is a common denominator of old age and essentially all chronic central nervous system (CNS) diseases [25]. J147 and CAD-31 both effectively reduce oxidative stress, with EC_50_ of 6 nM and 20 nM, respectively.
4. CAD-31 also prevents loss of energy metabolism that leads to neuron cell death in an in vitro ischemia model [26]. Its EC_50_ in this assay is 47 nM.
5. Finally, J147 and CAD-31 are able to block both extracellular and intracellular Aβ toxicity. Extracellular toxicity was assayed using rat hippocampal neurons with J147 having an EC_50_ of 15 nM, whereas CAD-31 is less potent in this assay (27 nM) [27]. Neither J147 nor CAD-31 binds to and directly inhibits Aβ_1–42_ aggregation using the thioflavin S assay (data not shown). Intracellular Aβ toxicity was assayed in the human neuron cell line MC65 that conditionally expresses the C99 fragment of the amyloid precursor protein. When the cells are induced to make Aβ, the cells die within 4 days. Both J147 and CAD-31 block this toxicity at around 10 nM. Therefore, CAD-31 has a repertoire of neuroprotective activities similar but not identical to that of its parent compound, J147.

### CAD-31 has an excellent pharmacological and safety profile

Before undertaking animal experiments, it is important to determine if a new drug candidate has a chance for further clinical development by determining its pharmacological properties of bioavailability and brain penetrance as well as predicted safety. Additional file 1: Table S1 shows that CAD-31 has good bioavailability in rats and excellent distribution to the brain when administered by gavage in corn oil. The maximum brain concentration at 20 mg/kg is about tenfold higher than the EC_50_ in the cell culture assays, with a brain-to-plasma ratio of 2.8 at 8 h. By multiple criteria used as initial steps to evaluate drug safety during the Investigational New Drug process, 10 μM CAD-31 showed no sign of acute toxicity or activity in the Ames test or micronucleus and hERG assays, and it did not block the activities of the five cytochrome P450 enzymes assayed (Additional file 1: Table S1). It did not interact with any known protein kinases or proteins in the lead profile screen panel of 60 brain ion channels, transporters, and receptors. At 10 μM, all of these assays were performed at greater than tenfold higher concentrations than their EC_50_ values in the cell culture assays, suggesting a good safety margin, and certainly disproved the medicinal chemistry dogma that compounds selected in the absence of an identified target will have poor safety profiles.

### CAD-31 reverses some aspects of cognitive dysfunction in old transgenic AD mice

J147 is one of the few compounds that has been tested in a mouse therapeutic paradigm to assay if the drug candidate can reverse preexisting memory deficits in old AD mice, and it was quite effective [2]. To determine if CAD-31 has similar properties, APPswe/PS1∆E9 mice were fed CAD-31 at 200 ppm in their chow (about 10 mg/kg/day) for 3 months starting at 10 months of age, a time when they had severe cognitive deficits and AD pathology [1, 2, 4, 7]. At this drug concentration in food, mice fed ad libitum (i.e., free-fed) had a maximum plasma and brain concentration of 82 nM and 53 nM, respectively (Additional file 1: Table S1). Nontransgenic littermates mice served as controls. At 13 months, the mice were run through a battery of cognitive tests and then killed for biochemical and molecular analysis.

**Fig. 2:**
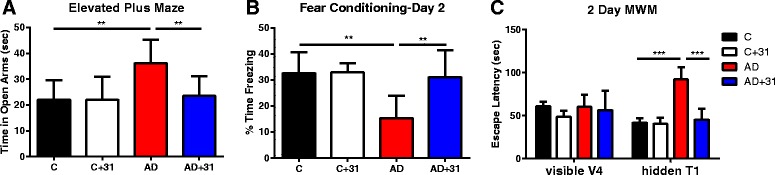
Female Alzheimer’s disease (AD) mice (*n* = 10 per group) aged 10 months old were divided into four groups as follows; wild-type (WT) control, WT treated with CAD-31, AD control, and AD treated with CAD-31. Treatment with CAD-31 (200 ppm in chow) continued for 3 months, followed by behavioral testing with several different memory assays. **a** Elevated plus maze. AD mice do not have a fear of open spaces and are less anxious than control mice, and CAD-31 corrected this deficit. **b** AD mice spent significantly less time freezing in response to the context compared with WT mice on day 2, demonstrating a significant deficit in hippocampus-related memory. Treatment with CAD-31 increased the amount of time AD mice spent freezing, suggesting a significant improvement in hippocampus-associated memory. **c** Two-day Morris water maze (MWM). There were four visible platform trials (V1––V4), where the last visible platform trial of a mouse was considered its posthabituation baseline. On day 2, 24 h following the last visible platform trial, mice were tested in a platform trial (T1). The data show that the AD mice had a very poor memory of the location of the platform that was greatly improved by CAD-31. One-way analysis of variance and Tukey’s post hoc test were used to determine the statistical significance of the behavioral responses. ** *p* < 0.01, *** *p* < 0.001

The open-field assay showed no differences between groups, demonstrating that CAD-31 has no overt toxicity in old mice (data not shown). The elevated plus maze measures the anxiety response of mice by comparing the time spent on the open arm with the time spent on the closed arm. AD mice tend to exhibit a disinhibition phenotype similar to that of patients with AD, and they spend more time on the open arm than on the closed arm. The data shown in Fig. 2a demonstrate that old transgenic AD mice do indeed spend more time on the open arm, a phenotype that was completely rescued to age-matched WT levels by feeding them CAD-31 for 3 months.

Fear conditioning was used to measure hippocampus-dependent associative learning. The mouse will freeze if it remembers and associates an environment with an aversive stimulus. The hippocampus is required for contextual fear memory. Thirteen-month-old AD mice spent significantly less time freezing in response to the context than WT control mice. The AD phenotype was rescued by treatment with CAD-31, as demonstrated by more time freezing in response to the context (Fig. 2b).

The 2-day water maze was used to analyze spatial navigational memory, which is impaired in APPswe/PS1∆E9 mice compared with their WT littermates. Briefly, a platform that is visible during training on day 1 is then submerged during testing on day 2, and mice use spatial cues on the wall around the pool to navigate to the platform. In Fig. 2c, visible V4 refers to the visible platform trial 4 (day 1) and is the last visible platform trial before testing, and therefore it represents the baseline. Results from day 1 indicate no defects in any of the mice in their ability to swim or see, because all have similar escape latencies.

During testing on day 2, the time it takes each mouse to find the hidden platform during trial 1 (hidden T1) is measured as escape latency. The AD mice take considerably longer than WT mice to find the hidden platform on day 2. CAD-31 significantly reduces the escape latency relative to AD mice to control levels (Fig. 2c), showing that CAD-31 can improve the spatial navigational memory in aged transgenic AD mice.

### RNA-seq analysis suggests that CAD-31 modulates inflammation, synaptic health, and metabolism

**Fig. 3:**
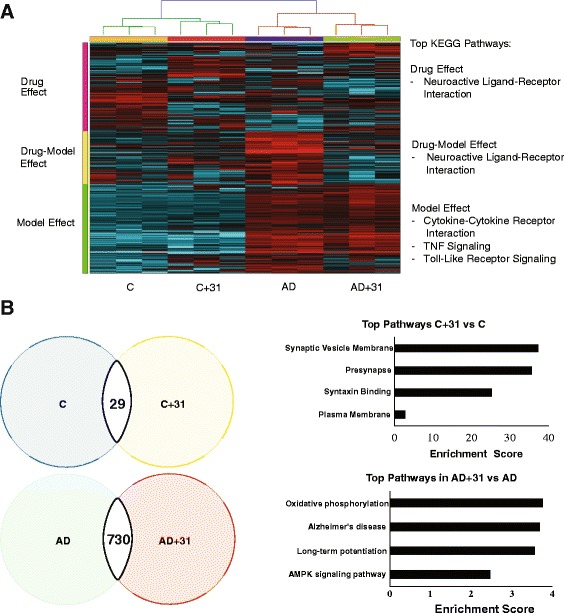
RNA-sequencing analysis shows pathways effected by CAD-31. A heat map of significant genes in hippocampal tissue between the control (C), CAD-31-treated control (C + 31), APPswe/PS1∆E9 mice (Alzheimer’s disease [AD]), and CAD-31-treated APPswe/PS1∆E9 mice (AD + 31) shows three distinct gene expression profiles. **a** Pathway analysis demonstrated that CAD-31 modulates neuroactive ligand-receptor interaction pathways in both the control and AD mice (drug effect and drug-model effect). The gene grouping generated by that AD model showed effects on proinflammatory pathways. **b** Advanced statistical analysis produced 29 genes differentially expressed between the control and CAD-31-treated control mice, as well as a 730-gene difference between the AD and CAD-31-treated AD mice. Pathway analysis revealed prosynaptic, metabolic, and disease-related pathways being changed by CAD-31 treatment. *n* = 3 per group. *AMPK* Adenosine monophosphate-activated protein kinase, *KEGG* Kyoto Encyclopedia of Genes and Genomes, *TNF* Tumor necrosis factor

To gain an initial insight into the mode of action of CAD-31, we assayed its effect on gene expression in the hippocampus using RNA-seq technology. Statistical software was used to determine the significant groupings and the differential expression of genes, and heat maps were established to identify and correlate changes in gene expression between groups. The heat maps showed that CAD-31 treatment had a strong effect and that there are three distinct sets of gene expression. There is a drug effect that is independent of the group, a drug effect uniquely in the AD model, and a model effect. Genes from these groups were processed through pathway enrichment analysis using the Kyoto Encyclopedia of Gene and Genomes (KEGG) algorithm to identify group- and drug-dependent pathways. Both the drug effect and the drug AD interaction groups showed a modulation of the neuroactive ligand-receptor interaction pathways. This group is broadly defined as molecular pathways modulated via the interaction of bioactive peptides and small molecules with receptors associated with cells of the nervous system. The AD model gene set identified modulation of cytokine-cytokine receptor interactions, tumor necrosis factor signaling, and Toll-like receptor signaling pathways, all related to inflammation (Fig. 3). Differential expression of genes was then determined between the control and drug-treated groups. There were 29 significant genes changed by CAD-31 in the WT model and 730 in the AD model (Fig. 3b). Pathway analysis demonstrated that CAD-31 has an effect on synapse formation in WT mice and revealed changes in oxidative phosphorylation, long-term potentiation, and AMPK signaling pathways in the AD model (Fig. 3b). We next determined if the modulation of any of these pathways could be confirmed by protein analysis.

### CAD-31 reduces inflammation and increases markers for neurogenesis and synapses

**Fig. 4:**
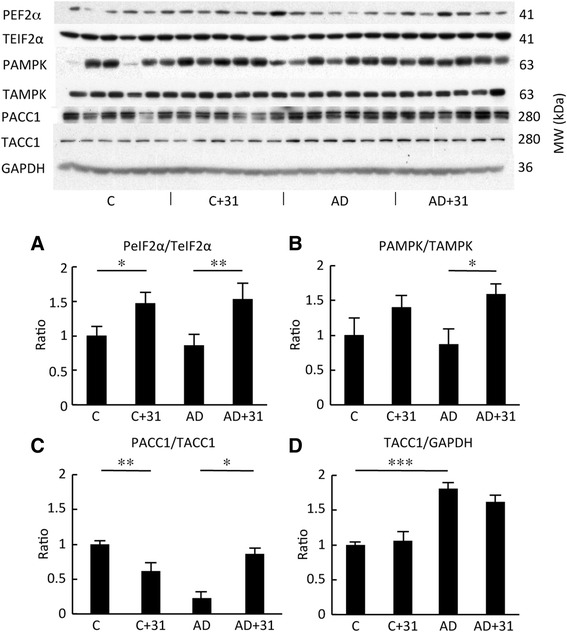
CAD-31 normalizes the expression of proteins from hippocampal tissue involved in endoplasmic reticulum stress and energy metabolism in old Alzheimer’s disease (AD) mice. **a** The ratio of phosphorylated eukaryotic initiation factor 2α (PEF2α; PeIF2α) to total eIF2α (TeIF2α). **b** The ratio of phosphorylated adenosine monophosphate-activated protein kinase (P-AMPK) to total AMPK (TAMPK). **c** Phosphorylated acetyl-coenzyme A carboxylase 1 (PACC1) to total ACC1 (TACC1). **d** Ratio of total ACC-1 to glyceraldehyde 3-phosphate dehydrogenase (GAPDH). *** *p* < 0.001; ** *p* < 0.01; * *p* < 0.05; *n* = 6 per group. *C + 31* CAD-31-treated control mice, *AD + 31* CAD-31-treated AD APPswe/PS1∆E9 mice, *MW* Molecular weight

Major pathways affected by CAD-31 that were identified in the RNA-seq data are those associated with AD, including proteotoxicity and AMPK signaling, both of which contribute to CNS inflammation. To gain insight into the mechanisms by which CAD-31 is functioning to reduce proteotoxicity in old AD mice, we examined key regulators of protein translation and the endoplasmic reticulum (ER) function. eIF2α is one of multiple proteins that regulates the rate of translation. The phosphorylation of threonine 51 reduces the rate of translation of a subset of proteins [28]. Our laboratories and others have shown that this event is very neuroprotective [29]. Figure 4a shows that CAD-31 stimulates eIF2α phosphorylation in both WT and old AD mice. Similarly, AMPK is the key regulator of protein translation via the inhibition of the mammalian target of rapamycin pathway, and it also induces autophagy, a process that is required for the removal of insoluble ubiquitinated proteins in the fly brain [30]. AMPK activation by phosphorylation on threonine 172 is enhanced by CAD-31 in both WT and old AD mice (Fig. 4b). ACC-1 catalyzes the synthesis of malonyl-coenzyme A (malonyl-CoA), the substrate for fatty acid synthesis. ACC-1 is phosphorylated and inactivated by AMPK [31]. Figure 4c shows that CAD-31 decreases the phosphorylation of ACC-1 in WT mice but greatly increases the depressed level of phosphorylation in AD mice. In contrast to phosphorylation, the total amount of ACC-1 protein is increased in AD mice, but it is not significantly reduced by CAD-31. With the exception of ACC-1 total protein, CAD-31 returns the expression and phosphorylation of these proteins to that of control WT mice in all cases. CAD-31 did not, however, alter the expression of the autophagy markers LC3 and P62, which were not changed between control and AD mice (data not shown).

**Fig. 5:**
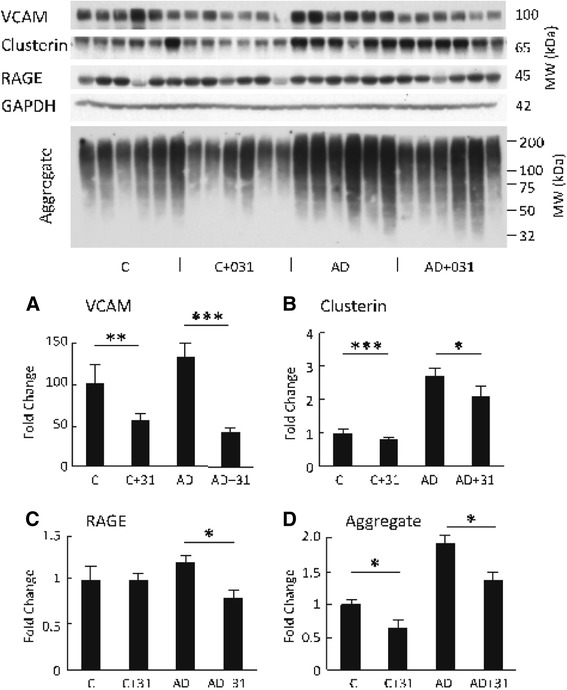
CAD-31 reduces markers for inflammation in hippocampal tissue. **a** Vascular cell adhesion molecule (VCAM). **b** Clusterin. **c** Receptor for advanced glycation endproducts (RAGE). **d** Radioimmunoprecipitation assay (RIPA) insoluble proteins. *** *p* < 0.001; * *p* < 0.05; *n* = 6 per group. *GAPDH* Glyceraldehyde 3-phosphate dehydrogenase, *C + 31* CAD-31-treated control mice, *AD + 31* CAD-31-treated Alzheimer’s disease APPswe/PS1∆E9 mice, *MW* Molecular weight

A major contributor to AD pathology is CNS inflammation. The gene expression data and the fact that CAD-31 activates AMPK, which in turn inhibits nuclear factor-κB activity, suggest that CAD-31 may reduce inflammation in the AD mice. To determine if CAD-31 is affecting inflammation in the old AD mice at the protein level, the proinflammatory VCAM, the proinflammatory RAGE, and clusterin were examined. Clusterin is a member of the heat shock family, is induced by inflammation, and is highly elevated in AD in humans and mice [32]. CAD-31 reduced the expression of all three proteins in AD mice (Fig. 5a–c).

The accumulation of both Aβ and aggregated, ubiquitinated nondisease proteins in the brain is also a common feature of AD [33]. Aggregates of Aβ within neurons induce a potent proinflammatory response from neurons themselves [34]. In flies, when detergent-insoluble, ubiquitinated, aggregated proteins are removed from the brain by increasing the rate of autophagy, the flies live longer, and when their removal is reduced by inhibiting autophagy, the flies’ lifespan is reduced [30]. When the synthetic precursor to CAD-31, called *CNB-001* (Fig. 1), is fed to APPswe/PS1∆E9-transgenic mice in a preventive paradigm, CNB-001 also reduces the accumulation of ubiquitinated, aggregated proteins in the brain [35]. In both 13-month-old WT and AD mice, there is an accumulation of RIPA-insoluble ubiquitinated proteins in the hippocampus that is reduced by CAD-31 (Fig. 5d), whereas the ubiquitinated proteins in the soluble fraction are unchanged (data not shown).

**Fig. 6:**
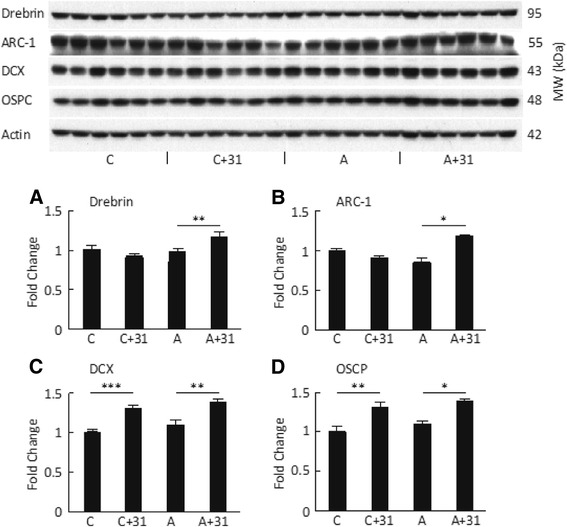
CAD-31 increases the expression of proteins associated with synapses, neurogenesis, and mitochondrial function in hippocampal tissue. **a** Drebrin. **b** Arc-1. **c** Doublecortin (DCX). **d** Oligomycin sensitivity-conferring protein (OSCP). *** *p* < 0.001; ** *p* < 0.01; * *p* < 0.05. *n* = 6 per group. *C + 31* CAD-31-treated control mice, *AD + 31* CAD-31-treated Alzheimer’s disease APPswe/PS1∆E9 mice, *MW* Molecular weight

In addition to revealing that CAD-31 had a robust effect on cognition, RNA-seq data showed that treatment with CAD-31 elevated the expression of many genes associated with synapse formation, long-term potentiation, and oxidative phosphorylation. We therefore examined markers for these pathways by Western blotting. Drebrin and Arc-1 are both synaptic proteins that are frequently used as surrogate markers for synaptic integrity [36, 37]. AD mice fed CAD-31 have increased expression of both proteins compared with AD mice not fed the compound (Fig. 6a and b). As in a preventive paradigm [4], CAD-31 increased the expression of the NPC marker DCX in the hippocampus (Fig. 6c). Finally, OSCP is a synapse-associated mitochondrial protein [38]. There is increased expression of OSCP in both control and AD mice (Fig. 6d) treated with CAD-31. Therefore, in most of these assays, CAD-31 maintains WT expression and thus WT homeostasis in the diseased animals. However, CAD-31 did not reduce the levels of either soluble or insoluble Aβ_1–42_ in this experimental paradigm (Additional file 2: Table S2).

Together, the results show that CAD-31 has the ability to rescue the cognitive decline and the disinhibition phenotype, as well as to reverse some aspects of the AD pathology seen in AD transgenic mice when administered at a late stage in the disease process, suggesting that CAD-31 has outstanding potential for the treatment of patients with AD.

### CAD-31 targets lipid metabolism

**Fig. 7:**
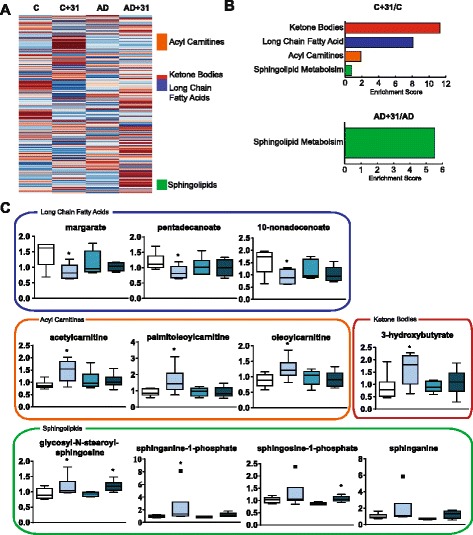
Metabolic analysis of plasma shows effects on lipid metabolism. Heat map of average levels of lipids from the plasma grouped by class. **a** General trends showed that CAD-31 had its largest effect on ketone bodies, acyl carnitine, long-chain fatty acids, and sphingolipids. **b** Metabolite enrichment scores were generated, and metabolite groups significantly changed were determined. Individual significantly changed metabolites from the enriched pathways were plotted. **c** The control (C), CAD-31-treated control (C + 31), Alzheimer’s mice (AD), and AD mice treated with CAD-31 (AD + 31) metabolite levels are color-coordinated to the enrichment graph. The control compared with the CAD-31-treated control and the AD vs CAD-31-treated AD group demonstrated general trends in lipid metabolism. One-way analysis of variance and Tukey’s post hoc test were used to determine statistical significance. * *p* < 0.05

Although transcriptional profiling is useful for predicting how drugs may affect the animals, they do not necessarily translate to relevant changes into the actual biochemistry of the tissue. Identifying changes in small-molecule metabolites is necessary to determine the physiological consequences of drug exposure as well as to find markers for target engagement [39]. To measure these metabolites, cortex and plasma samples from drug- and vehicle-treated AD and WT animals were analyzed by Metabolon, Inc., and organized by class. Metabolic profiling and pathway enrichment analysis of plasma suggested that lipid metabolism is a key aspect of mouse physiology that is modified by CAD-31. The top pathways modified in the plasma by CAD-31 in WT mice were ketone bodies, long-chain fatty acids, acyl carnitines, and sphingolipids (Fig. 7a and b). In contrast to the effect of CAD-31 on WT mice, the only significant pathways modified in the CAD-31-treated AD group compared with control AD mice were sphingolipids (Fig. 7b). Examples of metabolites from these pathways are shown in box plots (Fig. 7c).

**Fig. 8:**
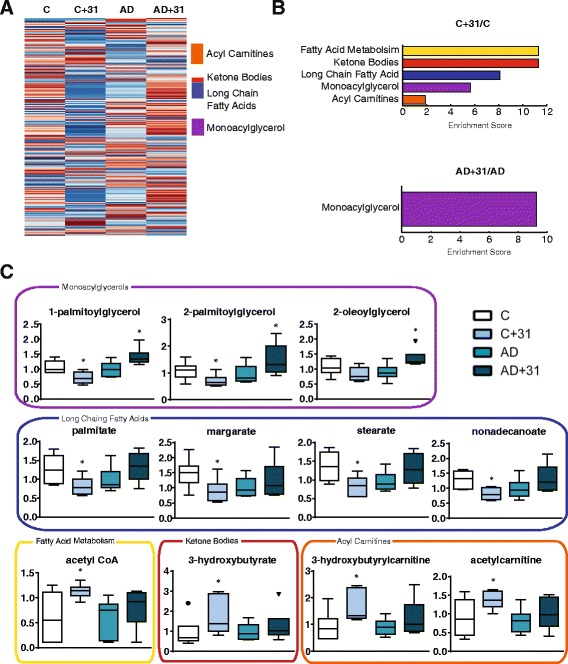
Metabolic analysis of the cortex in CAD-31-treated mice. **a** Heat map of average levels of lipids from the cortex, grouped by class. General trends showed that CAD-31 had a consistent effect on lipid levels. **b** Metabolite enrichment scores were generated, and metabolite groups significantly changed were determined. **c** Individual significantly changed metabolites from the enriched pathways are plotted. The control (C), CAD-31-treated control (C + 31), Alzheimer’s mice (AD), and AD mice treated with CAD-31 (AD + 31) metabolite levels are color-coordinated to the enrichment graph. The control compared with the CAD-31-treated control and the AD vs CAD-31-treated AD mice demonstrated general trends in lipid metabolism. One-way analysis of variance and Tukey’s post hoc test were used to determine statistical significance. * *p* < 0.05. *n* = 6 per group. *CoA* Coenzyme A

Brain metabolites in WT mice followed a similar pattern. The majority of differences occurred in lipid metabolism. Long-chain fatty acids and monoacylglycerols were decreased by CAD-31, whereas acetyl-CoA, ketone bodies, and acyl carnitines were increased by CAD-31, in WT mice (Fig. 8a and b). The only significant drug effect in the AD mice was an increase in monoacylglycerols (Fig. 8b). As with the plasma, these metabolic data suggest that CAD-31 is leading to a shift in mitochondrial lipid metabolism, reflected by increases in acyl carnitines, acetyl-CoA, and ketone bodies (Fig. 8c). Detailed metabolomic data are shown in the Additional file 3: Table S3 and Additional file 4: Table S4.

## Discussion

Because of the innate complexity of the CNS, essentially all of its associated diseases are multifactorial in the sense that there are a large number of toxicities that contribute to nerve cell death. Many, if not most, of these can be reproduced in cell culture assays, and compounds can be identified that inhibit these toxicities [4]. Using this approach, we have established six screening assays, identified natural products that are neuroprotective, and synthesized a large number of derivatives that have excellent medicinal chemical and pharmacological properties and are active with high potency in all of our assays [3, 40]. CAD-31 was selected from our chemical library on the basis of its neurogenic properties in human ES-derived NPCs [4]. Because of the modest change in structure of CAD-31 from its parent molecule J147, we predicted that the neuroprotective properties of J147 would be retained. Table 1 shows that when J147 was compared directly with CAD-31 in six assays, the two compounds had similar but not identical neuroprotective activities.

Several hundred compounds alter Aβ metabolism or improve behavioral deficits in AD transgenic mice using a preventive strategy in which the compound is given before disease onset [41], but none has translated to a viable therapeutic [42]. The reason for this may be that many of these compounds are tested only when administered before definitive pathology arises [43]. However, in humans, pathology is usually present at the time of diagnosis. To test the efficacy of CAD-31 in a more rigorous preclinical AD model, we treated mice using a therapeutic strategy that more accurately reflects the human symptomatic stage.

Tests that assess distinct aspects of human memory can be performed in rodents. Spatial memory is assessed using the Morris water maze [44], and hippocampus-dependent contextual memory can be analyzed by using a fear-conditioning assay [12]. In addition, the anxiety response of rodents can be measured using the elevated plus maze, an assay in which AD mice show a disinhibition phenotype [45, 46]. In the AD reversal treatment strategy described here, CAD-31 reduced the cognitive defect to a level found in WT mice of the same age.

CAD-31 treatment did not result in a significant effect on Aβ metabolism, because the levels of Aβ_1–42_ did not change in either the soluble or the insoluble fractions. This agrees with our early study which showed that CAD-31 had no effect on Aβ plaque density or size [4]. These Aβ data with the therapeutic model are supported by the observation that the protein levels of neither β-secretase nor amyloid precursor protein changed with CAD-31 treatment, whereas they did change in the preventive model, where there was a reduction in Aβ level [4].

RNA-seq analysis was conducted to determine the possible in vivo mechanisms of action of CAD-31. Significant changes in gene expression clearly clustered into three specific groups: a drug effect independent of the model, a drug effect only in the AD model, and an AD model group that is independent of drug treatment. The AD model grouping contained genes involved mainly in the inflammatory response, whereas the two drug-related groups displayed neuroactive ligand receptors. Further analysis identified genes differentially expressed between the groups. Whereas only 29 genes were significantly changed by CAD-31 in the WT mouse, pathway analysis suggests that these are involved in the modulation of synaptic function. CAD-31 displayed a greater effect in the AD model, where its effects on multiple pathways were identified, including AD, oxidative phosphorylation, long-term potentiation, and AMPK signaling. These data suggest that CAD-31 may be acting through anti-inflammatory, synapse-protective, and metabolic regulatory pathways. To verify these data, we measured the levels of key proteins in these pathways.

There is growing evidence that vascular inflammation may be directly involved in AD because inflammation and microvascular problems are ubiquitous features of the AD brain [47]. VCAM is a marker for vascular inflammation. VCAM expression is induced by reactive oxygen species and other pro-oxidants, and it is elevated in patients with AD [39, 48, 49]. VCAM is also elevated in 13-month-old APPswe/PS1∆E9 mice, and it is significantly reduced by CAD-31 along with another inflammation-related protein called *RAGE*. RAGE is elevated in patients with AD [50], and we show in the present study that CAD-31 reduces the level of RAGE well below the level in control mice.

Clusterin is a stress-induced chaperone molecule that is increased in AD and may be a biomarker for inflammation in the disease [51, 52]. But clusterin has a complex biology, and its functional association with AD is not clearly understood. In the present study, we show that CAD-31 significantly lowers the expression of clusterin, but unlike RAGE and VCAM, clusterin is not lowered to control levels.

The accumulation of intracellular Aβ and other aggregated insoluble proteins are likely triggers for inflammation and cell death in aging and in AD [33]. Moreover, the intraneuronal aggregated proteins very likely are the cause of ER stress in AD [53]. Like most animals, APPswe/PS1∆E9 transgenic mice accumulate aggregated, ubiquitinated proteins as they age [35], and here we show that CAD-31 reduces the amount of these proteins in old mice.

One way for cells to escape this form of stress is by the activation of the unfolded protein response (UPR). We have previously shown that the activation eIF2α, a protein that mediates this response, is neuroprotective both in vitro [24] and in vivo [1]. CAD-31 strongly stimulates the phosphorylation of eIF2α as well as AMPK, a central player in cellular metabolism that also mediates ER stress and autophagy [54].

There is a loss of synapses and dendritic structure in the 13-month-old APPswe/PS1∆E9 mice used in these experiments [2]. Two markers for synapses are drebrin and Arc-1. Drebrin and Arc are actin-binding synaptic proteins that have a role in synaptic plasticity [55–57]. CAD-31 increases the expression of both Arc and drebrin in AD mice. OSCP is a protein that is a subunit of mitochondrial F1F0-ATP synthase that is reduced in expression in AD [58]. Although it is not reduced in old AD mice, its expression is significantly elevated by CAD-31. These data are in line with the gene expression information showing that genes associated with ATP synthesis are upregulated by CAD-31. Together, these hippocampal protein expression data show that, when fed to APPswe/PS1∆E9 mice, CAD-31 reduces inflammation, enhances neuroprotective aspects of the UPR, and promotes synaptic structure.

Metabolic profiling was undertaken to identify potential biomarkers for CAD-31 therapy and to further understanding of the effect that CAD-31 has on small-molecule metabolism. In both the AD and WT groups, CAD-31 had the largest impact on lipid metabolites among the over 600 different molecules examined in the plasma and the cortex. In WT mice, CAD-31 increased levels of the ketone body 3-hydroxybutyrate and acyl carnitines, as well as acetyl-CoA levels in the brain. There was a decrease in fatty acids in both the brain and plasma. Ketone bodies, acyl carnitines, and acetyl-CoA are all associated with mitochondrial fatty acid metabolism, and they are all neuroprotective in various experimental paradigms [59–61]. The decrease in fatty acids, coupled with the increase in their byproducts, suggests that CAD-31 is increasing the rate of their oxidation, thereby enhancing energy metabolism. This mechanism may be able to compensate for the reduced rate of glucose metabolism associated with the AD brain [62]. CAD-31 also increases the levels of sphingolipids. Sphingolipid and sphingosine signaling are dysregulated in AD, and sphingosine-1-phosphate is a potential therapeutic for treating neurodegenerative diseases [63, 64].

The metabolic shift toward the breakdown of fatty acids induced by CAD-31 could be a possible mechanism of neuroprotection. It has been demonstrated that AMPK activation inhibits ACC-1, leading to a decrease in fatty acid synthesis and an increase in fatty acid β-oxidation [31]. The first step in fatty acid β-oxidation is the conjugation of a carnitine group for transport across the mitochondrial membrane. Once inside the mitochondria, the fatty acid is converted into acetyl-CoA, which can then be converted into ketone bodies for transport into the bloodstream. Ketone bodies are an important energy source for the CNS because long-chain fatty acids are unable to pass the blood-brain barrier. It has been reported that acetyl-CoA, acyl carnitines, and ketone bodies are dysregulated in AD, and all have shown promise as potential therapeutics. Metabolic regulation, such as through a ketogenic diet, has been successful in treating neurological disorders in the past, and clinical trials with this diet for AD are ongoing [65]. Experiments using dietary modification suggest that metabolic pathways are a legitimate target for treating AD [66]. CAD-31’s ability to upregulate three potentially therapeutic neuroprotective pathways is a possible mechanism for the reversal of AD pathology.

## Conclusions

**Fig. 9:**
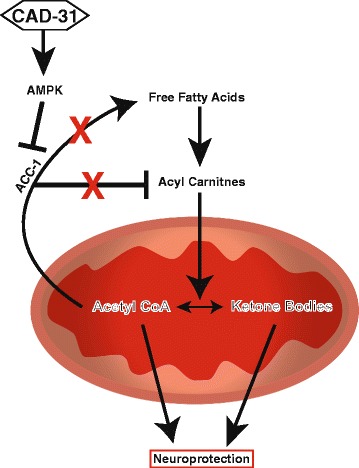
CAD-31 modifies lipid metabolism as a possible mechanism for neural protection. Free fatty acids are conjugated to carnitine for transport across the mitochondrial membrane. Once inside the mitochondria they can be metabolized to acetyl-coenzyme A (acetyl-CoA), which can then be transformed into ketone bodies. Acetyl-CoA can also be used by acetyl-coenzyme A carboxylase 1 (ACC-1) to form long-chain fatty acids. Adenosine monophosphate-activated protein kinase (AMPK) is able to phosphorylate ACC-1, inhibiting its function, which leads to an increase in available acetyl-CoA and ketone bodies. CAD-31 induces the activation of AMPK and modifies downstream genes. Acetyl-CoA and ketone bodies are able to be transported into the brain and have been shown to be neuroprotective

The data derived from the present study show that CAD-31 has therapeutic efficacy on cognitive and physiological parameters in a rigorous mouse model of AD in which the drug candidate was given to symptomatic mice for a relatively short period of time. CAD-31 reversed the cognitive deficiencies in the old AD mice to levels seen in age-matched control animals. The major physiological effects of CAD-31 were assayed by RNA-seq gene expression, metabolomics, and Western blotting. These data are summarized schematically in Fig. 9. Decreases in both fatty acids and monoacylglycerols could be due to the reduction of lipase activity or the increased catabolism of the fatty acids. CAD-31 causes an increase of both acyl carnitines and acetyl-CoA, which are precursors to fatty acid oxidation and ketone body synthesis. These data, together with the fact that genes associated with oxidative phosphorylation are elevated, suggest that CAD-31 is enhancing the use of free fatty acids for energy production. A common denominator of all of these pathways is AMPK. The phosphorylation and activation of AMPK and the resultant inhibition of ACC-1 block fatty acid synthesis, leading to increases in acetyl-CoA and ketone bodies. AMPK is a central player in many other aspects of energy metabolism and cell physiology related to stress and aging. These modifications in brain metabolism by CAD-31 create a more favorable physiology for the animal to deal with the AD-associated toxicities in this experimental paradigm and could have therapeutic efficacy late in human AD.

## Additional files

Additional file 1: Table S1. Pharmacokinetic and toxicology studies on CAD-31. (DOC 41 kb)

Additional file 2: Table S2. Soluble and insoluble Aβ_1–42_ (pg/mg protein). (DOC 28 kb)

Additional file 3: Table S3. Metabolomic profile of plasma. This table includes the significantly changed metabolites found in the plasma between the AD and control groups, as well as the vehicle- and CAD-31-treated groups. (XLSX 16 kb)

Additional file 4: Table S4. Metabolomic profile of cortex. This table includes the significantly changed metabolites found in the cortex between the AD and control groups, as well as the vehicle- and CAD-31-treated groups. (XLSX 17 kb)

## Abbreviations

ACC-1: Acetyl-coenzyme A carboxylase 1
AD: Alzheimer’s disease
AMPK: Adenosine monophosphate-activated protein kinase
ANOVA: Analysis of variance
Aβ: β-Amyloid
BDNF: Brain-derived neurotrophic factor
cDNA: Complementary DNA
CNS: Central nervous system
CoA: Coenzyme A
DCX: Doublecortin
EC_50_: Half-maximal effective concentration
eIF2α: Eukaryotic initiation factor 2α
ER: Endoplasmic reticulum
ES: Embryonic stem cell
FDR: False discovery rate
GAPDH: Glyceraldehyde 3-phosphate dehydrogenase
GSH: Glutathione
KEGG: Kyoto Encyclopedia of Genes and Genomes
MW: Molecular weight
MWM: Morris water maze
NPC: Neural precursor cell
OSCP: Oligomycin sensitivity-conferring protein
RAGE: Receptor for advanced glycation endproducts
RIPA: Radioimmunoprecipitation assay
RNA-seq: RNA sequencing
UPR: Unfolded protein response
VCAM: Vascular cell adhesion molecule
WT: Wild type

## References

[1] Chen Q, Prior M, Dargusch R, Roberts A, Riek R, Eichmann C (2011). A novel neurotrophic drug for cognitive enhancement and Alzheimer’s disease. PLoS One.

[2] Prior M, Dargusch R, Ehren JL, Chiruta C, Schubert D (2013). The neurotrophic compound J147 reverses cognitive impairment in aged Alzheimer’s disease mice. Alzheimers Res Ther.

[3] Prior M, Chiruta C, Currais A, Goldberg J, Dargusch R, Maher P (2014). Back to the future with phenotypic screening. ACS Chem Neurosci.

[4] Prior M, Goldberg J, Chiruta C, Farrokhi C, Kopynets M, Roberts AJ (2016). Selecting for neurogenic potential as an alternative for Alzheimer’s disease drug discovery. Alzheimers Dement.

[5] Currais A, Goldberg J, Farrokhi C, Chang M, Prior M, Dargusch R (2015). A comprehensive multiomics approach toward understanding the relationship between aging and dementia. Aging.

[6] Olin D, MacMurray J, Comings DE (2005). Risk of late-onset Alzheimer’s disease associated with BDNF C270T polymorphism. Neurosci Lett.

[7] Jankowsky JL, Slunt HH, Gonzales V, Savonenko AV, Wen JC, Jenkins NA (2005). Persistent amyloidosis following suppression of Aβ production in a transgenic model of Alzheimer disease. PLoS Med.

[8] Gulinello M, Gertner M, Mendoza G, Schoenfeld BP, Oddo S, LaFerla F (2009). Validation of a 2-day water maze protocol in mice. Behav Brain Res.

[9] Belzung C, Griebel G (2001). Measuring normal and pathological anxiety-like behaviour in mice: a review. Behav Brain Res.

[10] Lister RG (1987). The use of a plus-maze to measure anxiety in the mouse. Psychopharmacology.

[11] Thompson RF, Krupa DJ (1994). Organization of memory traces in the mammalian brain. Annu Rev Neurosci.

[12] Kim JJ, Jung MW (2006). Neural circuits and mechanisms involved in Pavlovian fear conditioning: a critical review. Neurosci Biobehav Rev.

[13] Bangasser DA, Waxler DE, Santollo J, Shors TJ (2006). Trace conditioning and the hippocampus: the importance of contiguity. J Neurosci.

[14] Maren S, Fanselow MS (1996). The amygdala and fear conditioning: has the nut been cracked?. Neuron.

[15] Rogan MT, LeDoux JE (1996). Emotion: systems, cells, synaptic plasticity. Cell.

[16] da Huang W, Sherman BT, Lempicki RA (2009). Systematic and integrative analysis of large gene lists using DAVID bioinformatics resources. Nat Protoc.

[17] Liu Y, Dargusch R, Maher P, Schubert D (2008). A broadly neuroprotective derivative of curcumin. J Neurochem.

[18] Lanni C, Stanga S, Racchi M, Govoni S (2010). The expanding universe of neurotrophic factors: therapeutic potential in aging and age-associated disorders. Curr Pharm Des.

[19] Abe K, Takayanagi M, Saito H (1990). Effects of recombinant human basic fibroblast growth factor and its modified protein CS23 on survival of primary cultured neurons from various regions of fetal rat brain. Jpn J Pharmacol.

[20] Rossler OG, Giehl KM, Thiel G (2004). Neuroprotection of immortalized hippocampal neurones by brain-derived neurotrophic factor and Raf-1 protein kinase: role of extracellular signal-regulated protein kinase and phosphatidylinositol 3-kinase. J Neurochem.

[21] Chao MV, Rajagopal R, Lee FS (2006). Neurotrophin signalling in health and disease. Clin Sci (Lond).

[22] Nakajo Y, Miyamoto S, Nakano Y, Xue JH, Hori T, Yanamoto H (2008). Genetic increase in brain-derived neurotrophic factor levels enhances learning and memory. Brain Res.

[23] Pezet S, Malcangio M (2004). Brain-derived neurotrophic factor as a drug target for CNS disorders. Expert Opin Ther Targets.

[24] Tan S, Schubert D, Maher P (2001). Oxytosis: a novel form of programmed cell death. Curr Topics Med Chem.

[25] Maher P, Parker L, Sies H, Eggersdorfer M, Cardenas E (2010). Modulation of multiple pathways involved in the maintenance of neuronal function by fisetin. Micronutrients and brain health.

[26] Maher P, Salgado KF, Zivin JA, Lapchak PA (2007). A novel approach to screening for new neuroprotective compounds for the treatment of stroke. Brain Res.

[27] Behl C, Davis JB, Lesley R, Schubert D (1994). Hydrogen peroxide mediates amyloid β protein toxicity. Cell.

[28] Pakos-Zebrucka K, Koryga I, Mnich K, Ljujic M, Samali A, Gorman AM (2016). The integrated stress response. EMBO Rep.

[29] Tan S, Somia N, Maher P, Schubert D (2001). Regulation of antioxidant metabolism by translation initiation factor-2α. J Cell Biol.

[30] Simonsen A, Cumming RC, Brech A, Isakson P, Schubert DR, Finley KD (2008). Promoting basal levels of autophagy in the nervous system enhances longevity and oxidant resistance in adult *Drosophila*. Autophagy.

[31] Wakil SJ, Abu-Elheiga LA (2009). Fatty acid metabolism: target for metabolic syndrome. J Lipid Res.

[32] Schrijvers EM, Koudstaal PJ, Hofman A, Breteler MM (2011). Plasma clusterin and the risk of Alzheimer disease. JAMA.

[33] Currais A, Fischer W, Maher P, Schubert D (2017). Intraneuronal protein aggregation as a trigger for inflammation and neurodegeneration in the aging brain. FASEB J.

[34] Currais A, Quehenberger O, Armando AM, Daugherty D, Maher P, Schubert D (2016). Amyloid proteotoxicity initiates an inflammatory response blocked by cannabinoids. NPJ Aging Mech Dis.

[35] Valera E, Dargusch R, Maher PA, Schubert D (2013). Modulation of 5-lipoxygenase in proteotoxicity and Alzheimer’s disease. J Neurosci.

[36] Ma L, Li Y, Wang R (2015). Drebrin and cognitive impairment. Clin Chim Acta.

[37] Minatohara K, Akiyoshi M, Okuno H (2016). Role of immediate-early genes in synaptic plasticity and neuronal ensembles underlying the memory trace. Front Mol Neurosci.

[38] Devenish RJ, Prescott M, Boyle GM, Nagley P (2000). The oligomycin axis of mitochondrial ATP synthase: OSCP and the proton channel. J Bioenerg Biomembr.

[39] Shin SY, Fauman EB, Petersen AK, Krumsiek J, Santos R, Huang J (2014). An atlas of genetic influences on human blood metabolites. Nat Genet.

[40] Chiruta C, Schubert D, Dargusch R, Maher P (2012). Chemical modification of the multitarget neuroprotective compound fisetin. J Med Chem.

[41] Zahs KR, Ashe KH (2010). ‘Too much good news’ – are Alzheimer mouse models trying to tell us how to prevent, not cure, Alzheimer’s disease?. Trends Neurosci.

[42] Wollen KA (2010). Alzheimer’s disease: the pros and cons of pharmaceutical, nutritional, botanical, and stimulatory therapies, with a discussion of treatment strategies from the perspective of patients and practitioners. Altern Med Rev.

[43] Savonenko A, Xu GM, Melnikova T, Morton JL, Gonzales V, Wong MP (2005). Episodic-like memory deficits in the APPswe/PS1dE9 mouse model of Alzheimer’s disease: relationships to β-amyloid deposition and neurotransmitter abnormalities. Neurobiol Dis.

[44] Morris RG, Garrud P, Rawlins JN, O’Keefe J (1982). Place navigation impaired in rats with hippocampal lesions. Nature.

[45] Meilandt WJ, Cisse M, Ho K, Wu T, Esposito LA, Scearce-Levie K (2009). Neprilysin overexpression inhibits plaque formation but fails to reduce pathogenic Aβ oligomers and associated cognitive deficits in human amyloid precursor protein transgenic mice. J Neurosci.

[46] Roberson ED, Scearce-Levie K, Palop JJ, Yan F, Cheng IH, Wu T (2007). Reducing endogenous tau ameliorates amyloid β-induced deficits in an Alzheimer’s disease mouse model. Science.

[47] Zenaro E, Piacentino G, Constantin G. The blood-brain barrier in Alzheimer’s disease. Neurobiol Dis. doi:10.1016/j.nbd.2016.07.007. 10.1016/j.nbd.2016.07.007 PMC5600438 27425887

[48] Cook-Mills JM, Marchese ME, Abdala-Valencia H (2011). Vascular cell adhesion molecule-1 expression and signaling during disease: regulation by reactive oxygen species and antioxidants. Antioxid Redox Signal.

[49] Ewers M, Mielke MM, Hampel H (2010). Blood-based biomarkers of microvascular pathology in Alzheimer’s disease. Exp Gerontol.

[50] Yan SD, Bierhaus A, Nawroth PP, Stern DM (2009). RAGE and Alzheimer’s disease: a progression factor for amyloid-β-induced cellular perturbation?. J Alzheimers Dis.

[51] Nuutinen T, Suuronen T, Kauppinen A, Salminen A (2009). Clusterin: a forgotten player in Alzheimer’s disease. Brain Res Rev.

[52] Won JC, Park CY, Oh SW, Lee ES, Youn BS, Kim MS (2014). Plasma clusterin (ApoJ) levels are associated with adiposity and systemic inflammation. PLoS One.

[53] Endres K, Reinhardt S (2013). ER-stress in Alzheimer’s disease: turning the scale?. Am J Neurodegener Dis.

[54] Kim H, Moon SY, Kim JS, Baek CH, Kim M, Min JY (2015). Activation of AMP-activated protein kinase inhibits ER stress and renal fibrosis. Am J Physiol Renal Physiol.

[55] Shim KS, Lubec G (2002). Drebrin, a dendritic spine protein, is manifold decreased in brains of patients with Alzheimer’s disease and Down syndrome. Neurosci Lett.

[56] Bramham CR, Alme MN, Bittins M, Kuipers SD, Nair RR, Pai B (2010). The *Arc* of synaptic memory. Exp Brain Res.

[57] Rudinskiy N, Hawkes JM, Betensky RA, Eguchi M, Yamaguchi S, Spires-Jones TL (2012). Orchestrated experience-driven *Arc* responses are disrupted in a mouse model of Alzheimer’s disease. Nat Neurosci.

[58] Beck SJ, Guo L, Phensy A, Tian J, Wang L, Tandon N (2016). Deregulation of mitochondrial F1FO-ATP synthase via OSCP in Alzheimer’s disease. Nat Commun.

[59] Maalouf M, Rho JM, Mattson MP (2009). The neuroprotective properties of calorie restriction, the ketogenic diet, and ketone bodies. Brain Res Rev.

[60] Jones LL, McDonald DA, Borum PR (2010). Acylcarnitines: role in brain. Prog Lipid Res.

[61] Zanelli SA, Solenski NJ, Rosenthal RE, Fiskum G (2005). Mechanisms of ischemic neuroprotection by acetyl-l-carnitine. Ann N Y Acad Sci.

[62] Chen Z, Zhong C (2013). Decoding Alzheimer’s disease from perturbed cerebral glucose metabolism: implications for diagnostic and therapeutic strategies. Prog Neurobiol.

[63] Couttas TA, Kain N, Daniels B, Lim XY, Shepherd C, Kril J (2014). Loss of the neuroprotective factor sphingosine 1-phosphate early in Alzheimer’s disease pathogenesis. Acta Neuropathol Commun.

[64] He X, Huang Y, Li B, Gong CX, Schuchman EH (2010). Deregulation of sphingolipid metabolism in Alzheimer’s disease. Neurobiol Aging.

[65] Wake Forest University Health Sciences. Effect of a Modified Ketogenic-Mediterranean Diet on Alzheimer’s Disease (BEAM). ClinicalTrials.gov identifier: NCT02984540. Registered on 30 Nov 2016.

[66] Bredesen DE (2014). Reversal of cognitive decline: a novel therapeutic program. Aging (Albany NY).

