# Supplementary material for: A novel Alzheimer’s disease drug candidate targeting inflammation and fatty acid metabolism
Source: Alzheimers Res Ther. 2017 Jul 14;9:50. doi: 10.1186/s13195-017-0277-3 (PMC5513091; doi:10.1186/s13195-017-0277-3)
Supplement: Supplementary file 1 — Pharmacokinetic and toxicology studies on CAD-31. (DOC 41 kb) [file 13195_2017_277_MOESM1_ESM.doc]

| **TABLE S1. Pharmacokinetic and Toxicology Studies on CAD-31** | |
| --- | --- |
|  | |
| **Pharmacokinetics** | **CAD-31 20 mg/kg** |
| PO Max Plasma | 8 hrs 1.7 μM |
| PO Max Brain | 8 hrs 3.0 μM |
| Bioavailability | 31 % |
| Brain/Plasma Ratio | 8 hrs 1.7 μM |
| t 1/2 | 4.3 hrs |
| Free-Fed ~10 mg/kg/day Plasma | 0.082 µM |
| Free-Fed ~10 mg/kg/day Brain | 0.053 µM |
|  | |
| **Safety** | **EC_50_** |
| Acute Toxicity (PO) | >2 g/kg |
| hERG | >10 μM |
| Ames | >10 μM |
| Micronucleus | >10 μM |
| Caco 2 Transport | High |
| CYP 3A4 | >10 μM |
| CYP 1A2 | >10 μM |
| CYP 2Cb | >10 μM |
| CYP 2D6 | >10 μM |
| CYP2C19 | >10 µM |
| Lead Profile Screen | >10 μM (all) |
| Protein Kinase Screen | >10 μM (all) |
|  | |
| The PK properties of CAD-31 were determined in rats, and safety parameters by the following CROs: Acute Toxicity, duration of observation one week post gavage (Salk); hERG (Eurofins); Ames (Pharmaron); Micronucleus (Cyprotex); Caco 2 Transport (Pharmaron); CYPs (Pharmaron); Lead Profile Screen (Eurofins); Protein kinase (DiscoverX). | |
