# Supplementary material for: A novel Alzheimer’s disease drug candidate targeting inflammation and fatty acid metabolism
Source: Alzheimers Res Ther. 2017 Jul 14;9:50. doi: 10.1186/s13195-017-0277-3 (PMC5513091; doi:10.1186/s13195-017-0277-3)
Supplement: Supplementary file 2 — Soluble and insoluble Aβ1–42 (pg/mg protein). (DOC 28 kb) [file 13195_2017_277_MOESM2_ESM.doc]

| **TABLE S2. Soluble and Insoluble Aβ_1-42_ (pg/mg protein)** | | |
| --- | --- | --- |
|  | **RIPA Soluble** | **RIPA Insoluble** |
| **AD** | 12.82 ± 1.447 | 3743 ± 396.4 |
| **AD+31** | 16.57 ± 3.359 | 4581 ± 489.3 |
| Levels of soluble and insoluble Aβ1-42 measured from the brain of vehicle and CAD-31 treated hAPPswe/PS1ΔE9 mice. | | |
